# Supplementary material for: PNLDC1, mouse pre‐piRNA Trimmer, is required for meiotic and post‐meiotic male germ cell development
Source: EMBO Rep. 2018 Feb 15;19(3):e44957. doi: 10.15252/embr.201744957 (PMC5836094; doi:10.15252/embr.201744957)
Supplement: Supplementary file 1 — Expanded View Figures PDF [file EMBR-19-e44957-s001.pdf]

Expanded View Figures

Figure EV1. Generation of *Pnldc1* mutant mice and phenotypes of *Pnldc1* exon 7 mutant mice (related to Fig 1).

A Body weights of adult control and *Pnldc1*$^{mt/mt}$ mice (n = 4).
B Scheme around exon 7 in *Pnldc1* mice and its targeted locus. PAM and gRNA-targeted sequences are underlined in black and green, respectively. A retrotransposon sequence was inserted with a 12-bp deletion at the gRNA-targeting region (red characters). Genotyping primers are labeled by black arrows.
C, D The inserted 836-bp retrotransposon sequence was confirmed by sequencing (C) and genotyping (D).
E Testicular sizes in adult control and *Pnldc1* exon 7 mutant mice. Scale bar: 2 mm.
F Hematoxylin- and eosin-stained sections of testes and epididymides of adult control and *Pnldc1* exon 7 mutant mice. Scale bar: 50 μm.

Source data are available online for this figure.

© 2018 The Authors

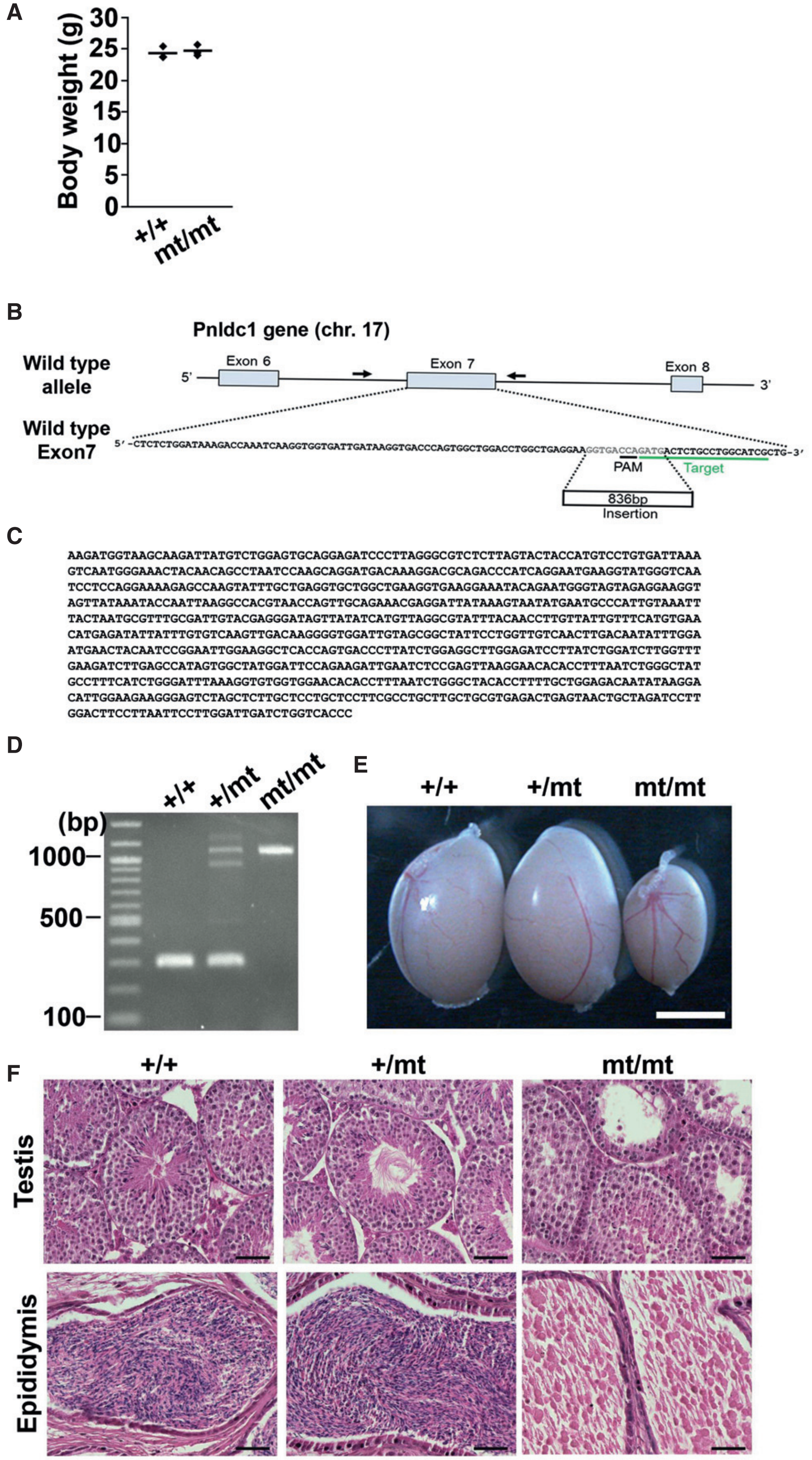

Figure EV1.

 © 2018 The Authors

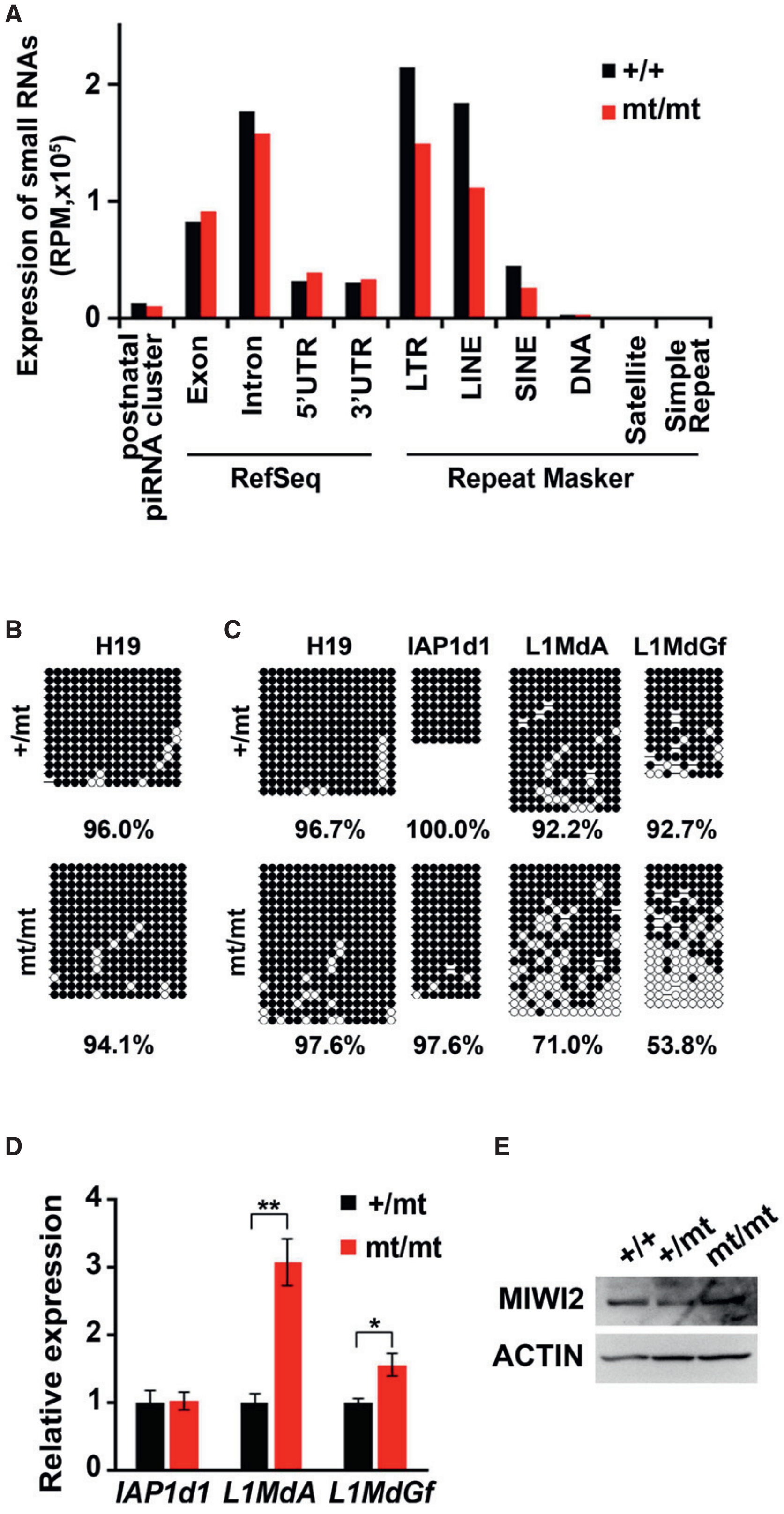

Figure EV2. Small RNAs and DNA methylation in *Pnldc1*$^{mt/mt}$ testes (corresponding to Fig 2).

A Expression of 24- to 50-nt small RNAs in control and *Pnldc1*$^{mt/mt}$ embryonic testes. The small RNAs from E16.5 control and *Pnldc1*$^{mt/mt}$ testes were analyzed after removal of rRNA and miRNA mapped reads by piPipes. The 24- to 50-nt small RNA reads were mapped to the mouse genome (mm9). Black and red bars indicate the control and *Pnldc1*$^{mt/mt}$ data, respectively.

B, C Bisulfite sequencing analysis of H19 DMR, IAP1d1, L1Md_A, and L1Md_Gf genes from purified 10-day-old male germ cells from control and *Pnldc1*$^{mt/mt}$ testes. DNA methylation of *Pnldc1*$^{mt/mt}$ mice (B) and exon 7 mutant mice (C) is shown.

D RT-qPCR analysis of IAP1d1, L1Md_A, and L1Md_Gf transcripts in testes from 14-day-old control and *Pnldc1*$^{mt/mt}$ mice. Expression levels were normalized to that of *β-actin*. Bars show mean ± SEM (n = 4). (P = 0.91 (IAP1d1), **P = 0.004 (L1Md_A), *P = 0.024 (L1Md_Gf) by t-test).

E Western blotting analysis of MIWI2 in E16.5 testes. β-ACTIN was used as a loading control.

Source data are available online for this figure.

© 2018 The Authors

Figure EV3. Analysis of MILI- and MIWI2-bound small RNAs of the control and Pnldc1$^{mt/mt}$ embryonic testes (corresponding to Fig 2).

A The RNAs that co-precipitated with MILI and MIWI2 were purified and separated by 15% denatured acrylamide gel electrophoresis after ^{32}P-end-labeling. Immunoprecipitated MILI and MIWI2 proteins were detected by WB with indicated antibodies.

B–E Length and nucleotide distributions of MILI- and MIWI2-bound small RNAs from the control and *Pnldc1$^{mt/mt}$* embryonic testes. MILI-bound small RNAs length distributions (B) and nucleotide distributions (C) are shown by bar graphs. MIWI2-bound small RNAs length distributions (D) and nucleotide distributions (E) are shown by bar graphs.

Source data are available online for this figure.

 © 2018 The Authors

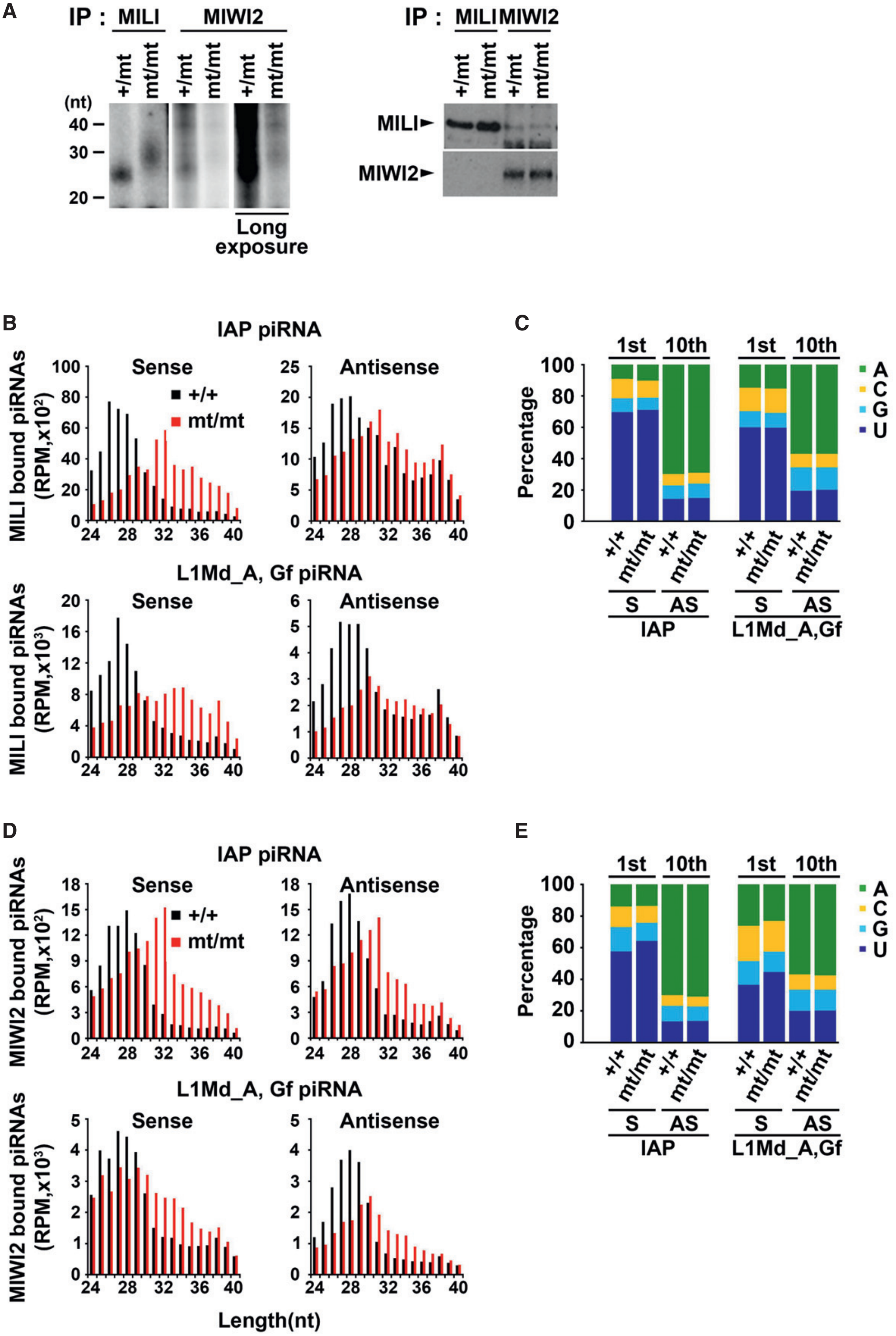
A
IP : MILI MIWI2
+/mt mt/mt +/mt mt/mt +/mt mt/mt
(nt)
40
30
20
Long exposure
IP : MILI MIWI2
+/mt mt/mt +/mt mt/mt
MILI
MIWI2
B
IAP piRNA
Sense
Antisense
+/+
mt/mt
MILI bound piRNAs (RPM,x10²)
L1Md_A, Gf piRNA
MILI bound piRNAs (RPM,x10³)
C
1st 10th 1st 10th
Percentage
A
C
G
U
S AS S AS
IAP
L1Md_A,Gf
D
IAP piRNA
MIWI2 bound piRNAs (RPM,x10²)
L1Md_A, Gf piRNA
MIWI2 bound piRNAs (RPM,x10³)
Length(nt)
E

Figure EV3.

Figure EV4. Post-natal small RNAs and nucleotide distribution around the mapped regions of embryonic LTR- and LINE-derived small RNAs (corresponding to Figs 3 and 4).

A Percentages of pachytene and pre-pachytene piRNAs.

B Expression of 24- to 50-nt small RNAs in control and *Pnldc1*$^{mt/mt}$ post-natal testes. Small RNAs from post-natal day 24 control and *Pnldc1*$^{mt/mt}$ testes were analyzed after the removal of rRNA and miRNA mapped reads by piPipes. The 24- to 50-nt small RNA reads were mapped to the mouse genome (mm9). Black and red bars show the control and *Pnldc1*$^{mt/mt}$ data, respectively.

C Nucleotide distribution around the small RNAs. Small RNA data corresponding to LTR (top) and LINE (bottom) from E16.5 control and *Pnldc1*$^{mt/mt}$ testes are shown. Asterisks (*) indicate strong T bias at the +1 position.

D ^{32}P-end-labeled synthesized oligo-RNAs with or without 2′-O-methylation at 3′ end were separated by 12% denatured acrylamide gels with or without β-elimination treatment.

Source data are available online for this figure.

© 2018 The Authors

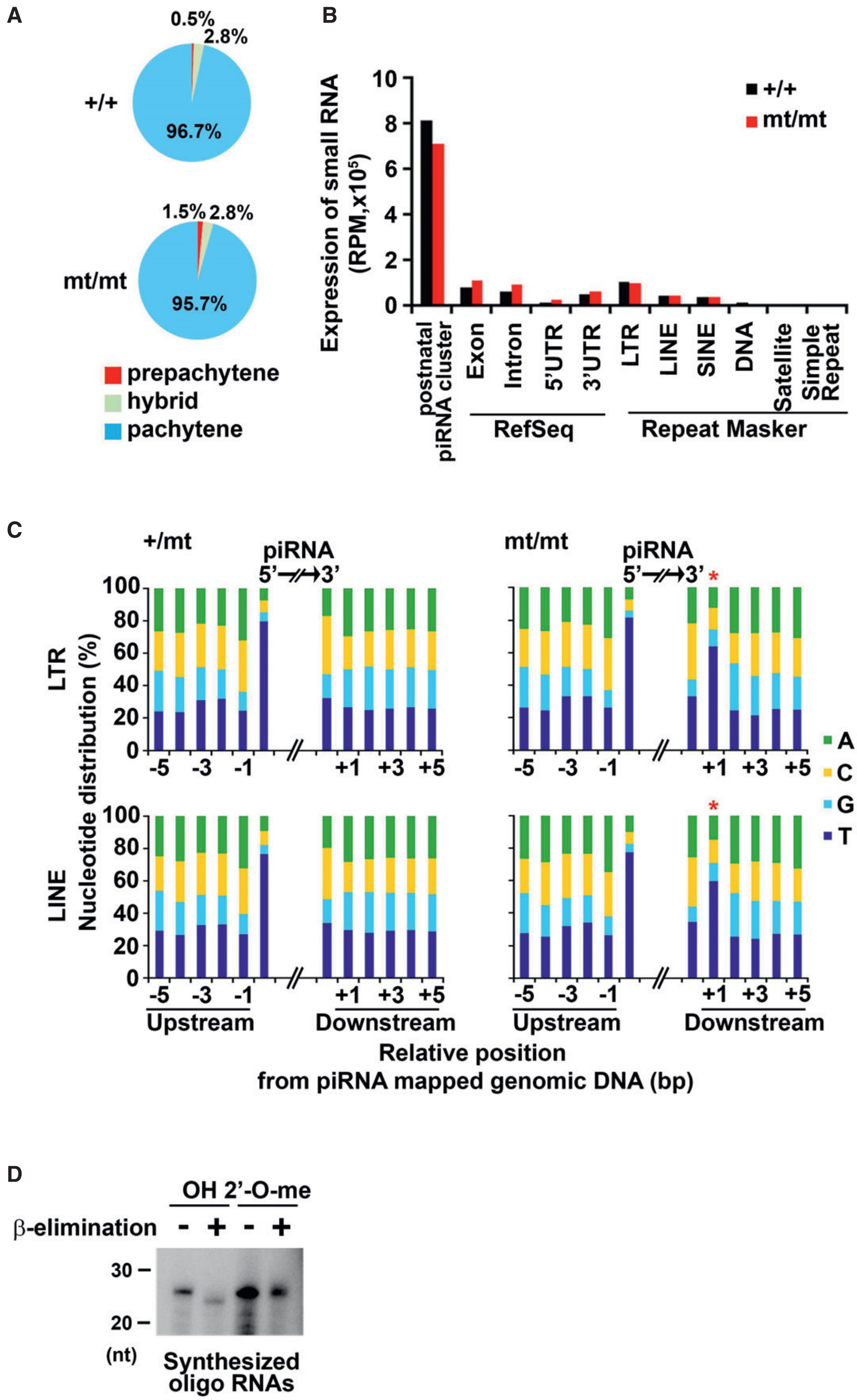
A
0.5%
2.8%
+/+
96.7%
1.5% 2.8%
mt/mt
95.7%
prepachytene
hybrid
pachytene
B
Expression of small RNA (RPM,x10⁵)
+/+
mt/mt
postnatal piRNA cluster
Exon
Intron
5'UTR
3'UTR
LTR
LINE
SINE
DNA
Satellite
Simple Repeat
RefSeq
Repeat Masker
C
+/mt
mt/mt
piRNA 5'→3'
LTR
LINE
Nucleotide distribution (%)
A
C
G
T
Upstream
Downstream
Relative position from piRNA mapped genomic DNA (bp)
D
OH 2'-O-me
β-elimination - + - +
30
20
(nt)
Synthesized oligo RNAs

Figure EV4.
