# Supplementary material for: PNLDC1, mouse pre‐piRNA Trimmer, is required for meiotic and post‐meiotic male germ cell development
Source: EMBO Rep. 2018 Feb 15;19(3):e44957. doi: 10.15252/embr.201744957 (PMC5836094; doi:10.15252/embr.201744957)
Supplement: Supplementary file 2 — Table EV1 [file EMBR-19-e44957-s002.docx]

**Table EV1. PCR primers for sgRNA targeting construct and genotyping**

| sgRNA | sequence |
| --- | --- |
| Exon3 Sense | 5'-AGGGTCTTCAGATACCACTCAGA-3' |
| Exon3 Antisense | 5'-AAACTCTGAGTGGTATCTGAAGAC-3' |
| Exon7 Sense | 5'-AGGGCGATGCCAGGCAGAGTCATC-3' |
| Exon7 Antisense | 5'-AAACGATGACTCTGCCTGGCATCG-3' |

| genotyping | sequence | Annealing  temperature | cycle |
| --- | --- | --- | --- |
| **Exon 3** |  |  |  |
| Forward | 5'-CCTTGCCTATGCAGTGAGATG-3' | 60℃ | 35 |
| Reverse | 5'-CGCCAAAGCTGGTTTTACCTAC-3' |  |  |
| **Exon 7**  Forward | 5'-AGTCACAGCGTTGAAAGCAC-3' | 60℃ | 35 |
| Reverse | 5'-GGCACACAGGAAAGAGAATCTG-3' |  |  |
