# Supplementary material for: PNLDC1, mouse pre‐piRNA Trimmer, is required for meiotic and post‐meiotic male germ cell development
Source: EMBO Rep. 2018 Feb 15;19(3):e44957. doi: 10.15252/embr.201744957 (PMC5836094; doi:10.15252/embr.201744957)
Supplement: Supplementary file 3 — Table EV2 [file EMBR-19-e44957-s003.docx]

**Table EV2. Primers for bisulfite sequencing analysis**

| PCR primer | sequence | Annealing  temperature | cycle |
| --- | --- | --- | --- |
| ***H19*** |  |  |  |
| 1^st^ Forward | 5'-GAGTATTTAGGAGGTATAAGAATT-3' | 56℃ | 30 |
| 1^st^ Reverse | 5'-ATCAAAAACTAACATAAACCCCT-3' |  |  |
| 2^nd^ Forward | 5'-GTAAGGAGATTATGTTTATTTTTGG-3' | 56℃ | 30 |
| 2^nd^ Reverse | 5'-CCTCATTAATCCCATAACTAT-3' |  |  |
| ***L1MdGf***  1^st^ Forward | 5'-GTTAGAGAATTTGATAGTTTTTGGAATAGG-3' | 56℃ | 10 |
| 1^st^ Reverse | 5'-CCAAAACAAAACCTTTCTCAAACACTATAT-3' |  |  |
| 2^nd^ Forward | 5'-TAGGAAATTAGTTTGAATAGGTGAGAGGT-3' | 56℃ | 30 |
| 2^nd^ Reverse | 5'-TCAAACACTATATTACTTTAACAATTCCCA-3' |  |  |
| ***L1MdA***  Forward | 5'-TTATTTTGATAGTAGAGTT-3' | 52℃ | 40 |
| Reverse | 5'-CRAACCAAACTCCTAACAA-3' |  |  |
| ***IAP1d1***  1^st^ Forward | 5'-GTTTGTAATGGTGGGAGAT-3' | 50℃ | 30 |
| 1^st^ Reverse | 5'-ATTCTAAAATAAAATATCCCTCC-3' |  |  |
| 2^nd^ Forward | 5'-AAATAAATTGTGGGAAGT-3' | 51℃ | 30 |
| 2^nd^ Reverse | 5'-CAAAAAAAACACCACAAACCAAAAT-3' |  |  |
