# Supplementary material for: PNLDC1, mouse pre‐piRNA Trimmer, is required for meiotic and post‐meiotic male germ cell development
Source: EMBO Rep. 2018 Feb 15;19(3):e44957. doi: 10.15252/embr.201744957 (PMC5836094; doi:10.15252/embr.201744957)
Supplement: Supplementary file 4 — Table EV3 [file EMBR-19-e44957-s004.docx]

**Table EV3. Sequencing primers for qPCR analysis**

| PCR primer | sequence | |  |  |
| --- | --- | --- | --- | --- |
| *Actin* Forward | 5'-CGGTTCCGATGCCCTGAGGCTCTT-3' |  |  |  |
| *Actin* Reverse | 5'-CGTCACACTTCATGATGGAATTGA-3' |  |  |  |
| *IAP 1d1* Forward | 5'-AACGCTGCTGCTTTAACTCC-3' |  |  |  |
| *IAP 1d1* Reverse | 5'-ATTGTTCCCTCACTGGCAAA-3' |  | |  |
| *L1MdA* Forward | 5'-CAGCTGAGTCGCCTGACAC-3' |  | |  |
| *L1MdA* Reverse | 5'-CTCTCCTTAGTTTCAGTGG-3' |  | |  |
| *L1MdGf* Forward | 5'-CTGTACCACCTGGGAACTGC-3' |  | |  |
| *L1MdGf* Reverse | 5'-TGCTGGCAAGCTCTCTTACA-3' |  | |  |
| *Mili* Forward | 5'- CCGCAAGGACAGAGAAGAAC-3' |  | |  |
| *Mili* Reverse | 5'- CTGCTCGTCCCAGTGGTAAC-3' |  | |  |
| *Miwi* Forward | 5'- CCTCAAGTCAGTCGGGAGAGG-3' |  | |  |
| *Miwi* Reverse | 5'- CAGGCCACTGCTGTCATAGATG-3' |  | |  |
