# Supplementary material for: PNLDC1, mouse pre‐piRNA Trimmer, is required for meiotic and post‐meiotic male germ cell development
Source: EMBO Rep. 2018 Feb 15;19(3):e44957. doi: 10.15252/embr.201744957 (PMC5836094; doi:10.15252/embr.201744957)
Supplement: Supplementary file 5 — Table EV4 [file EMBR-19-e44957-s005.docx]

**Table EV4. Summary of small RNA sequence**

|  | Embryonic day 16 | | Postnatal day 24 | |
| --- | --- | --- | --- | --- |
|  | Control | Pnldc1mt/mt | Contrl | Pnldc1mt/mt |
| Total mapped reads (20-50 nt) | 4,075,314 | 3,058,627 | 11,986,359 | 7,611,232 |
| piRNA cluster (24-50nt) |  |  |  |  |
| prepachytene | 43,499 | 25,888 | 50,600 | 79,786 |
| hybrid | 4,526 | 2,905 | 274,983 | 152,373 |
| pachytene | 5,281 | 2,919 | 9,401,830 | 5,154,063 |
| Refseq (24-50nt) |  |  |  |  |
| Gene | 947,874 | 658,817 | 1,552,509 | 1,323,406 |
| Exon | 337,178 | 281,038 | 932,420 | 811,393 |
| Intron | 721,776 | 481,876 | 708,718 | 668,213 |
| 5UTR | 131,074 | 119,371 | 174,600 | 195,457 |
| 3UTR | 126,032 | 104,164 | 599,508 | 470,055 |
| RepeatMasker (24-50nt) | 1,932,832 | 988,192 | 2,389,050 | 1,450,825 |
| DNA | 14,418 | 10,673 | 122,624 | 51,878 |
| SINE | 183,398 | 79,493 | 439,409 | 273,719 |
| LINE | 752,260 | 342,281 | 491,549 | 304,095 |
| LTR | 871,049 | 457,159 | 1,251,324 | 738,514 |
| Satellite | 1,549 | 741 | 1,657 | 1,615 |
| Simple_repeat | 2,470 | 1,318 | 12,799 | 6,762 |
