# Supplementary figures and images for: PNLDC1, mouse pre‐piRNA Trimmer, is required for meiotic and post‐meiotic male germ cell development
Source: EMBO Rep. 2018 Feb 15;19(3):e44957. doi: 10.15252/embr.201744957 (PMC5836094; doi:10.15252/embr.201744957)

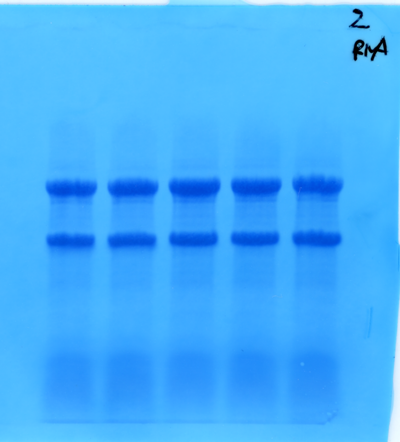

Supplement: Supplementary file 9 — Source Data for Figure 2 [file EMBR-19-e44957-s007.zip › 44957_Source_Data_for_Figure2/44957_Source_Data_for_Fig2E-A.tif]

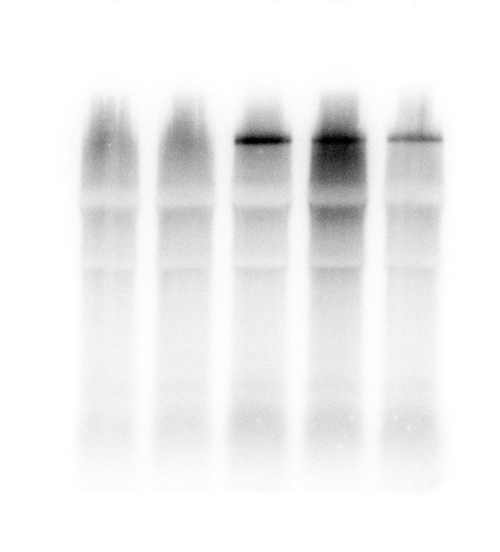

Supplement: Supplementary file 9 — Source Data for Figure 2 [file EMBR-19-e44957-s007.zip › 44957_Source_Data_for_Figure2/44957_Source_Data_for_Fig2E-A_North.tif]

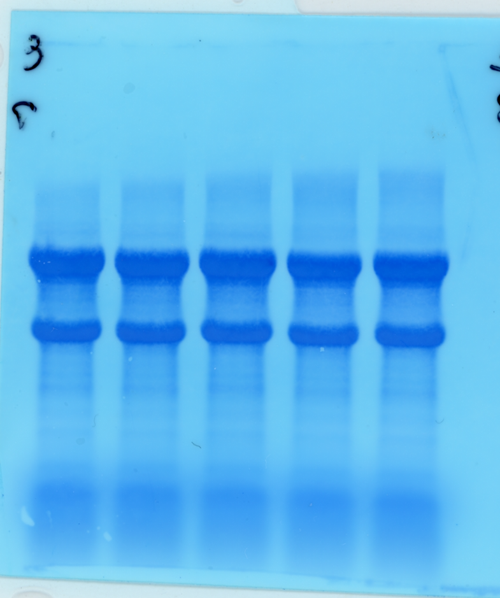

Supplement: Supplementary file 9 — Source Data for Figure 2 [file EMBR-19-e44957-s007.zip › 44957_Source_Data_for_Figure2/44957_Source_Data_for_Fig2E-T_.tif]

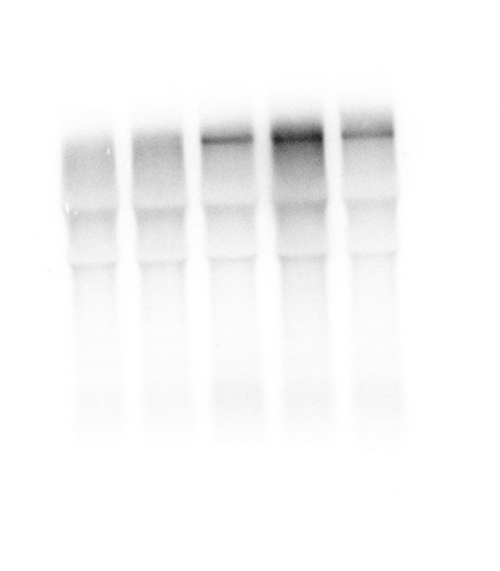

Supplement: Supplementary file 9 — Source Data for Figure 2 [file EMBR-19-e44957-s007.zip › 44957_Source_Data_for_Figure2/44957_Source_Data_for_Fig2E-T_North.tif]

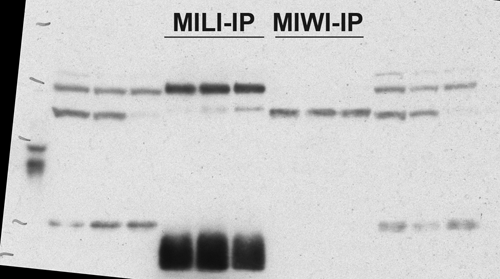

Supplement: Supplementary file 10 — Source Data for Figure 4 [file EMBR-19-e44957-s008.zip › 44957_Source_Data_for_Figure4/44957_Source_Data_for_Fig4A_IP.tif]

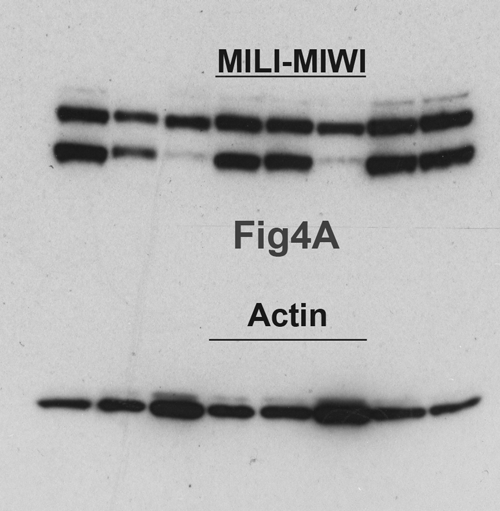

Supplement: Supplementary file 10 — Source Data for Figure 4 [file EMBR-19-e44957-s008.zip › 44957_Source_Data_for_Figure4/44957_Source_Data_for_Fig4A_MILI_Actin.tif]

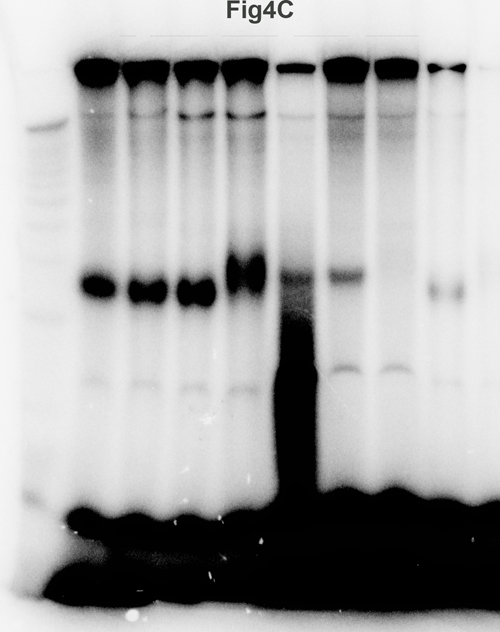

Supplement: Supplementary file 10 — Source Data for Figure 4 [file EMBR-19-e44957-s008.zip › 44957_Source_Data_for_Figure4/44957_Source_Data_for_Fig4C.tif]

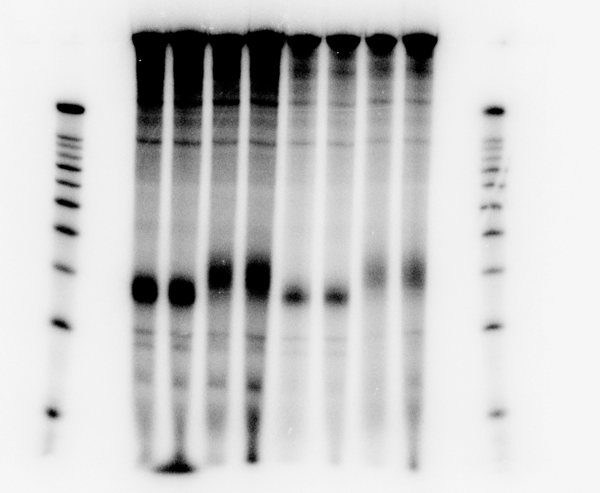

Supplement: Supplementary file 10 — Source Data for Figure 4 [file EMBR-19-e44957-s008.zip › 44957_Source_Data_for_Figure4/44957_Source_Data_for_Fig4D.tif]
